# Update to the Vitamin C, Thiamine and Steroids in Sepsis (VICTAS) protocol: statistical analysis plan for a prospective, multicenter, double-blind, adaptive sample size, randomized, placebo-controlled, clinical trial

**DOI:** 10.1186/s13063-019-3775-8

**Published:** 2019-12-04

**Authors:** Christopher J. Lindsell, Anna McGlothlin, Samuel Nwosu, Todd W. Rice, Alex Hall, Gordon R. Bernard, Laurence W. Busse, E. Wesley Ely, Alpha A. Fowler, David F. Gaieski, Jeremiah S. Hinson, Michael H. Hooper, James C. Jackson, Gabor D. Kelen, Mark Levine, Greg S. Martin, Richard E. Rothman, Jonathan E. Sevransky, Kert Viele, David W. Wright, David N. Hager

**Affiliations:** 1 Department of Biostatistics, Vanderbilt University Medical Center, Nashville, TN USA; 2 Berry Consultants, LLC, Austin, TX USA; 3 Division of Pulmonary & Critical Care, Department of Medicine, Vanderbilt University Medical Center, Nashville, TN USA; 4 Department of Emergency Medicine, Emory University, Atlanta, GA USA; 5 Grady Memorial Hospital, Atlanta, GA USA; 6 Division of Pulmonary, Allergy, Critical Care, and Sleep Medicine, Department of Medicine, Emory University, Atlanta, GA USA; 7 Critical Illness, Brain Dysfunction, and Survivorship (CIBS) Center, Vanderbilt University Medical Center, Nashville, TN USA; 8 Tennessee Valley Veteran’s Affairs Geriatric Research Education Clinical Center (GRECC), Nashville, TN USA; 9 Division of Pulmonary Disease & Critical Care Medicine, Department of Internal Medicine, The VCU Johnson Center for Critical Care and Pulmonary Research, Virginia Commonwealth University School of Medicine, Richmond, VA USA; 10 Department of Emergency Medicine, Sidney Kimmel Medical College, Thomas Jefferson University, Philadelphia, PA USA; 11 Department of Emergency Medicine, Johns Hopkins University, Baltimore, MD USA; 12 Division of Pulmonary & Critical Care Medicine, Department of Medicine, Eastern Virginia Medical School and Sentara Healthcare, Norfolk, VA USA; 13 Department of Psychiatry, Vanderbilt University Medical Center, Nashville, TN USA; 14 Molecular & Clinical Nutrition Section, Intramural Research Program, National Institute of Diabetes and Digestive and Kidney Diseases, National Institutes of Health, 10 Center Drive, Bethesda, MD USA; 15 Division of Pulmonary, Allergy, Critical Care, and Sleep Medicine, Department of Medicine, Emory University, Emory Critical Care Center, Atlanta, GA USA; 16 Division of Pulmonary & Critical Care Medicine, Department of Medicine, Johns Hopkins University, 1800 Orleans Street, Suite 9121, Baltimore, MD 21287 USA

**Keywords:** Statistical analysis plan, Adaptive sample size, Vitamin C, Thiamine, Steroids, Sepsis, Septic shock

## Abstract

**Background:**

Observational research suggests that combined therapy with Vitamin C, thiamine and hydrocortisone may reduce mortality in patients with septic shock.

**Methods and design:**

The Vitamin C, Thiamine and Steroids in Sepsis (VICTAS) trial is a multicenter, double-blind, adaptive sample size, randomized, placebo-controlled trial designed to test the efficacy of combination therapy with vitamin C (1.5 g), thiamine (100 mg), and hydrocortisone (50 mg) given every 6 h for up to 16 doses in patients with respiratory or circulatory dysfunction (or both) resulting from sepsis. The primary outcome is ventilator- and vasopressor-free days with mortality as the key secondary outcome. Recruitment began in August 2018 and is ongoing; 501 participants have been enrolled to date, with a planned maximum sample size of 2000. The Data and Safety Monitoring Board reviewed interim results at *N* = 200, 300, 400 and 500, and has recommended continuing recruitment. The next interim analysis will occur when *N* = 1000.

This update presents the statistical analysis plan. Specifically, we provide definitions for key treatment and outcome variables, and for intent-to-treat, per-protocol, and safety analysis datasets. We describe the planned descriptive analyses, the main analysis of the primary end point, our approach to secondary and exploratory analyses, and handling of missing data. Our goal is to provide enough detail that our approach could be replicated by an independent study group, thereby enhancing the transparency of the study.

**Trial registration:**

ClinicalTrials.gov, NCT03509350. Registered on 26 April 2018.

## Introduction

Sepsis is a devastating condition for which there are few effective therapies. With the exception of antimicrobials and vasopressors, pharmaceutical interventions have failed to improve patient outcomes in clinical trials [1, 2]. As a result, contemporary treatment remains limited to early appropriate antibiotics, fluid resuscitation, hemodynamic support, and control of infection [3]. Recently, combination therapy with vitamin C, thiamine, and steroids has garnered interest following an observational cohort study, with historical controls, that suggested an absolute reduction in mortality of over 30% [4]. The potential benefit of this treatment regimen is biologically plausible [5]. We designed and implemented a multicenter, double-blinded, randomized, placebo-controlled, adaptive sample size clinical trial to investigate the efficacy of this combination therapy in patients with sepsis.

The protocol for the Vitamin C, Thiamine, and Steroids in Sepsis (VICTAS) trial (NCT03509350) was previously reported [6]. Briefly, initial enrollment was planned to include up to 500 participants and continue to a maximum of 2000 participants if no stoppage rules are triggered. Stoppage rules were defined a priori and will determine the final number of participants enrolled in the trial. Participants are aged 18 years or older with suspected or confirmed sepsis who are either admitted to, or awaiting admission to, an intensive care unit (ICU). The presence of sepsis is evidenced by: 1) the ordering of blood cultures and the administration of at least one antimicrobial agent; and 2) acute respiratory and/or cardiovascular organ dysfunction that is attributed to the sepsis event. Randomization to either treatment or control must occur within 24 h of onset of the first qualifying organ dysfunction, and study drugs (or placebos) must be started within 4 h of randomization. To date, 43 sites have been activated to enroll patients, and 501 patients have been enrolled.

Details of the statistical design and adaptations, including sample size justification, interim analysis plans, and the approach to multiplicity during interim looks were published with the protocol. Interim analyses have occurred at *N* = 200, *N* = 300, *N* = 400 and *N* = 500, and the Data and Safety Monitoring Board has recommended continuing recruitment at each time point. Here, we describe in detail the statistical analysis plan that will be used when the trial ends. This includes definitions of key treatment and outcome variables, definitions of the analysis datasets, our approach to handling missing data, our approach to analyzing primary, secondary, exploratory and safety end points, and the inclusion of sensitivity analyses to explore the robustness of our findings.

## Treatment arms

Participants are randomized 1:1 to receive either intervention or control, defined as follows.

### Intervention

The intervention consists of intravenous vitamin C (1.5 g), thiamine hydrochloride (100 mg), and hydrocortisone sodium succinate (50 mg) administered within 4 h of randomization and then every 6 h thereafter. The intervention continues until either the patient is discharged from the ICU or 16 drug administrations have been completed (96 h), whichever is first. On days when a patient is treated with open-label hydrocortisone at a dose ≥200 mg/day (or equivalent), study drug 3 (hydrocortisone succinate) is withheld. If a subsequent daily medication check during the treatment period reveals that open-label hydrocortisone has been decreased to <200 mg/day (or equivalent), study drug 3 (hydrocortisone succinate) will be administered.

### Control

The control condition involves matching placebos administered intravenously within 4 h of randomization and then every 6 h thereafter. Placebo administration continues until either the patient is discharged from the ICU or 16 drug administrations have been completed (96 h), whichever is first. On days when a patient is treated with open-label hydrocortisone at a dose ≥200 mg per day (or equivalent), study drug 3 (placebo) is withheld. If a subsequent daily medication check during the treatment period reveals that open-label hydrocortisone has been decreased to <200 mg/day (or equivalent), study drug 3 (placebo) will be administered.

## End points

### Primary end point

**Fig. 1:**
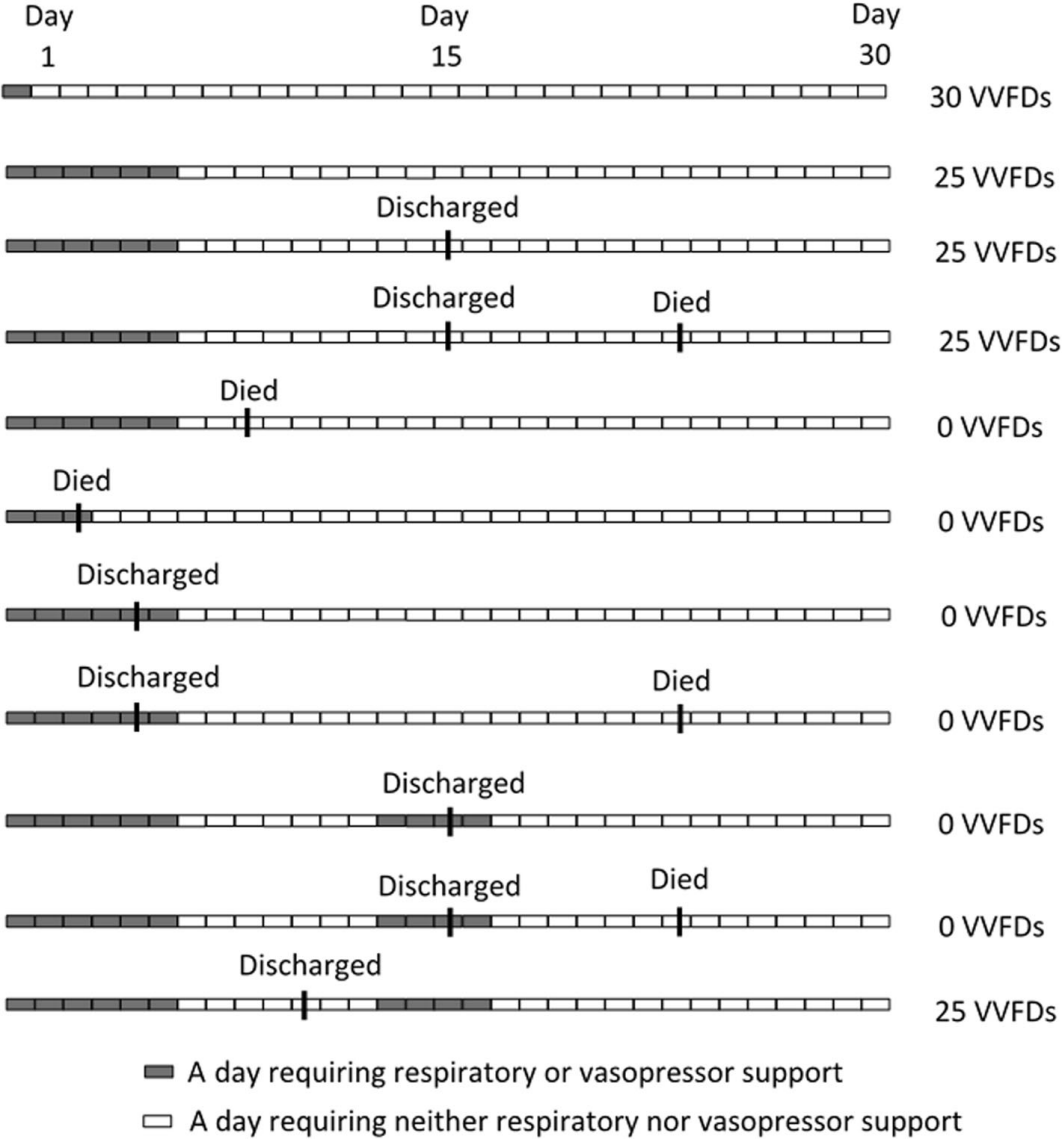
Illustration of the calculation of ventilator- and vasopressor-free days (VVFDs) under various scenarios

The primary end point for this trial is ventilator- and vasopressor-free days (VVFD) on day 30 following midnight of the randomization day. This is computed as a backwards count of consecutive whole days free of both respiratory and vasopressor support between day 30 and the most recent use of either respiratory or vasopressor support (Fig. 1). Note that days free of respiratory and vasopressor support that occur between periods with support do not count towards VVFD. The day of randomization is day 0 and the next calendar day is day 1. Day 0 will not contribute to the count because participants must require respiratory support or vasopressors to be enrolled. For a day to count as free of both respiratory and vasopressor support, a patient cannot receive any of the following on that calendar day: 1) mechanical ventilation via an endotracheal tube or tracheostomy tube; 2) noninvasive positive-pressure ventilation with supplemental oxygen; 3) high-flow nasal cannula at ≥40 l/min with a fraction of inspired oxygen ≥0.4; or 4) norepinephrine, epinephrine, vasopressin, dopamine, phenylephrine, angiotensin II, or other vasopressor agents (not including pure inotropes) at any dose for any duration.

Participants in need of respiratory or vasopressor support on day 30 will be assigned zero VVFDs. Participants who die before day 30 will also be assigned zero VVFDs. For participants alive but not observed to day 30 (e.g., participants who are discharged or transferred to another facility), the last observed status will be carried forward. Thus, if the participant was last seen on respiratory or vasopressor support, it is assumed they remained that way until day 30 and will thus be assigned zero VVFDs. If the participant was last seen not requiring respiratory or vasopressor support, it is assumed they stayed that way until day 30 and the unobserved days will count as VVFDs.

We note that it is possible a patient who is discharged might experience a subsequent hospitalization during which respiratory or vasopressor support is provided. However, we are unlikely to have knowledge of such events and they will not be counted against the VVFD for the original enrollment; status at discharge for the first hospitalization will be carried forward.

The count of VVFDs is an ordinal variable, with death or the requirements for respiratory or vasopressor support resulting in lower scores, and sustained absence of respiratory and vasopressor support resulting in higher scores.

### Key secondary end point

The secondary end point for this trial is 30-day mortality. The 30 days begins at midnight on the day of randomization (day 0). A death within 30 days will count towards this end point. Death is a binary variable. As with VVFDs, we will use the last observed value carried forward when a patient is discharged prior to day 30. That is, a patient discharged alive will be assumed alive at day 30.

### Additional exploratory end points

Additional exploratory end points are:

1. Change in the Sequential Organ Failure Assessment (SOFA) score between baseline and day 4 [7].
2. A binary variable indicating mortality between the time of randomization and 180 days following midnight on the day of randomization.
3. A binary variable indicating mortality between the time of randomization and departure from the ICU.
4. Length of ICU stay, measured in days, from midnight following randomization to the day of departure from the ICU; a partial day will count as a whole day.
5. Length of hospital stay, measured in days, from midnight following randomization to the day of departure from the hospital; a partial day will count as a whole day.
6. Renal replacement-free days at day 30, computed as a backwards count of consecutive whole days free of renal replacement therapy beginning at day 30 from midnight of the day of randomization (i.e., calculation will follow the same rules as VVFD).
7. ICU delirium, measured as the number of whole days alive and free of both delirium and coma between midnight on the day of randomization and day 5. Delirium and coma are assessed using the confusion assessment method for the ICU (CAM-ICU) [8, 9]. All coma- and delirium-free days count towards this end point regardless of whether they are consecutive or not. If multiple assessments are done on a single day, all assessments must be free of delirium and coma for the day to count towards the end point.
8. Neurocognitive outcomes at 180 days measured among survivors using the following instruments:
  - Attention (Wechsler Adult Intelligence Scale IV: digit span) [10]
  - Delirium (telephone confusion assessment method) [11]
  - Executive Functioning (Hayling test) [12]
  - Language (controlled oral word association test) [13]
  - Orientation (telephone interview for cognitive status) [14]
  - Memory (Wechsler Memory Scale IV: paragraph recall) [15]
  - Reasoning (Wechsler Adult Intelligence Scale IV: similarities) [15]
  - Activities of daily living (Katz) [16]
  - Employment (employment questionnaire)
  - Instrumental activities of daily living (functional activities questionnaire) [17]
  - Depression (Beck Depression Inventory II) [18]
  - Post-traumatic stress disorder checklist for the Diagnostic and Statistical Manual 5th revision [19]
  - EuroQol, five dimensions [20]

### Safety end points

Safety end points to be included in the safety analysis for this trial include all potentially associated adverse events (PAAEs). The prespecified PAAEs are:

- Nephrolithiasis
- Hemolysis
- Hypersensitivity reactions
- Injection site reactions

In addition, we will report other PAAEs that are not listed above. All PAAEs will be characterized by the nonstudy clinical care team managing the patient. Due to the nature and clinical course of patients with sepsis and septic shock, a substantial number of adverse events are expected among participants, including but not limited to:

- Death
- Renal failure
- Respiratory failure
- Heart failure
- Pneumonia or other/new infection
- Deep vein thrombosis or pulmonary embolism
- Complications related to ICU procedures
- Arrhythmia
- Delirium
- Bowel ischemia
- Ileus
- Leukopenia or leukocytosis
- Anemia or thrombocytopenia
- Coagulopathy (disseminated intravascular coagulation)
- Hypoglycemia
- Electrolyte abnormalities

These adverse events are common in sepsis and septic shock and are thus not expected to reflect the safety of the treatment regimen. Conversely, absence of these events is expected to contribute to efficacy outcomes, and several are included as efficacy end points. There is no plan to summarize or report these events to characterize safety.

## Design considerations

The trial design, including adaptations, stopping rules and power considerations, was previously described [6]. It is briefly reviewed here for context.

### Randomization

Participants are randomized in a 1:1 ratio to receive either intervention or placebo. Randomization uses permuted small blocks of random size, stratified within site. No other stratification or control for imbalance is being used. The randomization schema is deployed via the central investigational pharmacy.

### Adaptations and stopping

The trial was designed to detect a moderate effect on the primary end point of VVFDs, while allowing early stoppage if a very large effect is observed on the secondary end point of mortality. Early interim analyses were performed to compute the predicted probability of success on the mortality end point with *N* = 200, 300, and 400 enrolled participants. Interim analyses include all data, monitored and unmonitored, for completed participants, as well as information on the number of patients enrolled who do not yet have outcomes available. Efficacy stopping rules, but not futility stopping rules, were in place for these early interim analyses. If a sufficiently large effect was observed, accrual would have been stopped, enrolled participants would have been followed for outcomes and the primary analysis would have focused on the mortality end point.

Because the trial has proceeded beyond *N* = 400, additional interim analyses will be conducted at *N* = 500, 1000, and 1500 based on both VVFDs and mortality. Both futility and efficacy rules are defined for these interims. Once a stopping rule has been triggered or when 2000 patients have been recruited, accrual will stop, all enrolled participants will be followed for outcomes and the primary analysis will focus on the VVFD end point.

### Power and sample size

If the intervention truly causes a mortality difference of 20%, study power is approximately 99%, and the trial would likely have stopped before 500 participants were enrolled with a very high probability (>95%) of success. For a true mortality difference of 5% and true average improvement of 0.6 days free of respiratory or vasopressor support for participants that do not die, the power of the study is approximately 95%. The primary outcome assessed if the study stops when *N* = 500, 1000, 1500, or 2000 participants is VVFDs. The overall type I error rate for the trial is controlled at 2.5%. Thus, the early interim analyses at *N* = 200, 300, and 400 were designed to conservatively spend alpha so that 2.4% remains for the analyses of 500 or more enrolled participants. Having exceeded 500 participants, a more moderate effect on mortality remains possible, and the trial is planned to continue beyond 500 participants.

## Definition of analysis sets

### Intent-to-treat analysis set

All randomized participants will be included in the intent-to-treat analysis set. The intent-to-treat participants will be used for all primary, secondary, and other efficacy analyses. In these analyses, participants will be classified according to the treatment to which they were randomized, regardless of what treatments or how many study treatments were given.

Participants who withdraw consent will be included in the intent-to-treat analysis set. If, at the time consent was withdrawn, the participant gave consent for observation of outcomes then observed outcomes will be used. Otherwise, the last observed value will be carried forward.

### Per-protocol analysis set

All participants who are included in the intent-to-treat analysis set who correctly receive at least four doses of assigned study treatment (all three components of the study drug or placebo, adjusted for open-label steroids) and did not incur any major protocol deviations or violations will be included in the per-protocol analysis set. In this analysis, participants will be classified according to the treatment they received. Major protocol deviations or violations will be identified prior to the unblinding of the study for the final analysis and will include:

- Found to violate any inclusion or exclusion criterion
- Condition adjudicated not to be sepsis
- Received one or more doses of the unassigned study treatment
- Study hydrocortisone (or placebo) not adjusted for use of open-label steroids.
- Other protocol deviations classified as ‘major’ by a majority vote of the Executive Committee, who shall be blinded at the time of the vote

### Safety analysis set

Participants who are randomized and receive at least one administration of study treatment will be included in the safety analysis set. If a subject received both placebo and active treatment, they will be considered as having received active treatment. All other participants will be classified as not having received active treatment.

## Analysis

### Timing of analysis

Once a decision to stop the trial has been made, the primary analysis may proceed after all enrolled participants have completed the 30-day follow-up, 30-day data have been monitored, and 30-day data are declared query free. All data up to and including day 30 will be locked at this time. Analysis of additional efficacy end points and long-term outcomes will proceed after all enrolled participants have completed 180-day follow-up, the 180-day data have been monitored, the 180-day data are declared query free, and the remainder of the database has been locked.

### Blinding

Trial investigators and research teams are blinded to treatment assignment. There are two groups of study statisticians, one of which is performing the interim analyses and one of which is conducting the primary study analyses. Neither group of statisticians is blinded. This statistical analysis plan was drafted prior to the first interim analysis and prior to unblinding.

### Descriptive analysis

Using data pooled across all sites, the study sample will be characterized based on demographic and clinical variables measured at randomization, unless otherwise indicated. Specifically, the following variables will be described:

1. Age (years)
2. Race (African American, Caucasian, other)
3. Ethnicity (Hispanic or Latino, not Hispanic or Latino, or not reported)
4. Sex (male or female)
5. Education (less than high school, high school or general education diploma, some college)
6. Body mass index (kg/m^2^)
7. Medical history (yes, no):
  - Diabetes
  - Cardiovascular disease
  - Neurological disease
  - Respiratory illness
  - Current cancer
8. Eligibility criterion (respiratory support, vasopressor support, both)
9. Source of admission (emergency department, intermediate care (or step-down unit), floor, other)
10. Admission reason (sepsis, other medical, urgent surgical (necrotizing soft tissue, bowel obstruction, bowel ischemia, burn, trauma), other surgical)
11. Baseline vital measurements (closest measurement prior to time of randomization):
  - Heart rate (beats per minute)
  - Systolic blood pressure (mmHg)
  - Diastolic blood pressure (mmHg)
  - Mean arterial pressure (mmHg)
  - Respiration rate
  - Temperature (°C)
12. Baseline laboratory values (closest measurement prior to time of randomization):
  - White blood cell count (K/mm^3^)
  - Platelets (K/mm^3^)
  - Hemoglobin (g/dL)
  - Lactate (mmol/L)
  - Creatinine (mg/dL)
13. Baseline severity:
  - Acute Physiology and Chronic Health Evaluation II score (continuous score) [21]
  - SOFA score (continuous score)
  - CAM-ICU (delirium present or absent)
14. Infection, using final available value:
  - Infection source (lung, blood or vascular access, urinary tract, intra-abdominal, skin or soft tissue, central nervous system, bone or joint, other, unknown; if no confirmed source available, use final presumed source)
  - Gram-positive organism
  - Gram-negative organism
  - Fungal infection
  - Organism not identified
  - Other infection
  - Unknown infection

Categorical variables will be described using frequencies and proportions. Continuous variables will be described using mean and standard deviation, as well as median and interquartile range (IQR). The sample will be described overall and stratified by group assignment according to the intent-to-treat principle. No statistical testing will be done to compare characteristics between groups.

### Main analysis

The main analysis will be a simple comparison between the two treatment groups according to the intent-to-treat principle. If the study had stopped before *N* = 500, the first analysis would have been based on mortality. Because the study has proceeded to *N* = 500, the first analysis will be based on VVFDs.

#### VVFDs

A Wilcoxon rank-sum test (i.e., Mann–Whitney *U* test) will be used to compare VVFDs between treatment groups using a one-sided alpha of 0.022. As described in the adaptive design report, this threshold controls the type 1 error accounting for multiple analyses at *N* = 500, 1000, 1500 and 2000 [6]. If the sample size had been *N* < 500, the VVFD end point would have only been tested if the mortality end point had been successful.

#### Mortality

Had the study stopped prior to *N* = 500, the mortality end point would have been tested first with a chi-square test using a one-sided alpha = 0.001 (i.e., 0.1%). Because the study has reached *N* = 500, and is planned to continue enrollment, mortality will be compared between treatment groups only if there is a difference observed on VVFDs. In this case, a one-sided alpha of 0.024 will be used.

We note that the Wilcoxon rank-sum test is equivalent to a proportional odds model with one binary predictor, and the Chi-square test is equivalent to the test of significance of a binary predictor in a logistic regression model. For simplicity in comparing the unadjusted analyses to the adjusted analyses described later, we will also report the odds ratios with 95% confidence intervals.

#### Description of end points

End points will be described using median and IQR for VVFDs and frequency and percentages for mortality. The distribution of VVFDs will be described using histograms. Mortality point estimates will be reported with 95% confidence intervals. Descriptions will be given overall, and for each treatment group. Differences in median VVFDs will be computed with 95% confidence intervals. Similarly, differences in proportions for mortality will be calculated with 95% confidence intervals.

### Sensitivity analysis

Our sensitivity analyses are not designed to preserve type I error rates, but rather to explore possible sources of bias that might inform interpretation of the main analysis. As such, all sensitivity analyses we will use a two-sided alpha of 0.05. We will also emphasize the magnitude and confidence intervals of differences over statistical significance.

#### Per-protocol analysis

We will duplicate our main analysis using the per-protocol dataset.

#### Missingness

Due to the method of using last value carried forward to assign unobserved outcomes, there will be no missingness on the primary outcomes for the main analysis. We will conduct one sensitivity analysis in which we will replicate the main analysis but include only those participants with observed outcomes.

#### Steroids

Since participants in either arm can receive open-label steroids, we will replicate the primary analysis excluding those who were treated with open-label steroids in the placebo group.

### Safety analysis

This study is not designed to test safety. No statistical comparison of safety will be done. Safety end points will be reported in tabular format, grouped by whether the participant received any active treatment or not.

### Adjusted analysis

We will use two approaches to estimate treatment effects adjusted for covariates. Generalized linear mixed models will be used to estimate the conditional effect of treatment with site as a random effect. Generalized estimating equations will be used to estimate the marginal effect of study treatment. Mortality will be modeled assuming a logit link function. A proportional odds model will be specified for VVFDs. Models will consider baseline variables as listed in the descriptive analysis. Multiple imputation based on predictive mean matching will be used to overcome any missingness in covariates. Restricted cubic splines will be used for addressing potential nonlinearities in the association between continuous variables and outcomes. Some collinearity is expected among baseline variables. If we observe substantial collinearity, such as a correlation greater than 0.6 or a variance inflation factor greater than 2.5, we will use a principal components analysis approach where the first principle component of the correlated variable group will be included in the model. Interaction terms will not be considered in the main adjusted analysis. Models will be constructed based on the intent-to-treat principle. Effect sizes and overall fit will be emphasized for selecting the final model; statistical significance alone will not be used to decide which variables are included in the models. Graphical approaches will be used to explore fit; for example, residual plots can be used to identify issues with the proportional odds assumptions and with nonlinearity. The Wald minus df can also be plotted to explore importance of individual predictors in the models. The fitted models will then be applied to the per-protocol analysis set in a sensitivity analysis. In addition, if any multiple imputation were to be required, the fitted models will be applied to complete cases.

### Differential treatment effects and subgroup analysis

Using the fitted models from the principal adjusted analysis, we will evaluate the interaction between treatment group assignment and each of the following variables: sex, race, ethnicity, age, admission reason, source of admission, infection, baseline illness severity, baseline lactic acid, and eligibility criteria. Interactions will not be tested together within the same model, but will be tested one by one. If any interaction achieves a *P* value ≤0.2, we will report treatment effects within each subgroup defined by that interaction variable.

### Analysis of exploratory end points

A number of exploratory end points have been specified. We will proceed with exploring the effect of treatment on these end points in a similar manner as for the primary and secondary end points:

- End points will be described as medians and IQR or frequencies and proportions; histograms will be generated for ordinal variables
- Binary variables will be compared using a chi-square test and ordinal variables will be compared using a Wilcoxon rank-sum test.
- Differences and 95% confidence intervals of differences between study arms will be computed
- Exploratory end points will be modeled with adjustment for baseline covariates
- Differential treatment effects will be evaluated, and consequent subgroup effects reported

All analyses of exploratory end points will proceed under the intent-to-treat principle. A *P* value of 0.05 will be used, although emphasis will be placed on effect sizes. We do not expect exploratory end points to be continuous and normally distributed. However, any outcome variable that meets these criteria may be compared between treatment groups using student’s *t* test and a linear link function will be used for modeling purposes. It is possible that exploratory end points may be missing. Analyses of exploratory end points in the presence of missing outcomes will proceed using complete case analysis. A sensitivity analysis using multiple imputation for missing outcomes will be conducted.

## Summary

The analyses described here are those necessary to answer the trial’s primary question of whether combined treatment with vitamin C, thiamine and steroids is more effective than placebo in increasing days alive and free from respiratory and vasopressor support and reducing mortality in patients with sepsis.

Beyond our analysis exploring the effect of treatment on primary, secondary, and exploratory end points, we expect there to be multiple additional exploratory analyses conducted. It is not possible to predetermine the nature of such analyses, particularly as a rich biospecimen repository is being developed as a component of this study. However, we are committed to preserving rigor and reproducibility and will prespecify each subsequent analysis in the context of the specific question to be answered, cognizant of bias and missingness in the data.

## Data Availability

A de-identified dataset from participants in the VICTAS trial will be made publicly available about 1 year after publication of the primary manuscript.

## Abbreviations

CAM-ICU: Confusion assessment method for the intensive care unit
ICU: Intensive care unit
IQR: Interquartile range
PAAE: Potentially associated adverse event
SOFA: Sequential Organ Failure Assessment
VICTAS: Vitamin C, Thiamine and Steroids in Sepsis
VVFD: Vasopressor- and ventilator-free days
